# Association of Stress and Musculoskeletal Pain With Poor Sleep: Cross-Sectional Study Among 3,600 Hospital Workers

**DOI:** 10.3389/fneur.2018.00968

**Published:** 2018-11-21

**Authors:** Jonas Vinstrup, Markus Due Jakobsen, Joaquin Calatayud, Kenneth Jay, Lars Louis Andersen

**Affiliations:** ^1^National Research Centre for the Working Environment, Copenhagen, Denmark; ^2^Sport Sciences, Department of Health Science and Technology, Aalborg University, Aalborg, Denmark; ^3^Exercise Intervention for Health Research Group, Department of Physiotherapy, University of Valencia, Valencia, Spain; ^4^The Carrick Institute for Graduate studies, Institute of Clinical Neuroscience and Rehabilitation, Cape Canaveral, FL, United States

**Keywords:** health care personnel, sleep, stress, pain, working environment

## Abstract

**Background:** While acute stress and pain are part of our inherent survival mechanisms, persistent stress and pain can negatively impact health and well-being. This may also lead to poor sleep and thus a lack of recovery. This study investigated the influence of stress and musculoskeletal pain on sleep quality.

**Methods:** A total of 3,593 Danish hospital workers replied to a questionnaire about work and health. Pain intensity was evaluated using subjective values as an average of 9 body parts. Stress was assessed using the full version of Cohen's Perceived Stress scale. Sleep quality was rated using 3 questions on sleep characteristics. Associations between stress and pain (mutually adjusted predictors) and sleep (outcome) were modeled using binary logistic regression controlling for gender, age, education, BMI and smoking.

**Results:** The risk ratio of moderate stress (compared to no/low stress) on poor sleep was 1.27 (CI 1.26–1.29), whereas the risk ratio of high stress on poor sleep was 1.87 (CI 1.83–1.91). Similarly, for pain, the risk ratio of moderate pain (compared to no/low pain) on poor sleep was 1.18 (95% CI 1.16–1.19), whereas the risk ratio of a high pain score on poor sleep was 1.48 (95% CI 1.44–1.52).

**Conclusion:** This study demonstrates that both stress and musculoskeletal pain are associated with poor sleep among hospital workers. Hospital management should consider implementing strategies for preventing stress and musculoskeletal pain to improve the overall health and workability among hospital workers.

## Introduction

Sleep serves vital biological functions including, but not limited to; physiological processes, learning, memory, health and cognition (1, 2). Consequently, sleep deprivation yields numerous detrimental effects on the organism and even mild sleep deprivation negatively affects pain sensitivity in healthy adults (3, 4). For example, sleep deprivation has been shown to be an important risk factor of decreased immunity (5), to increase the pro-inflammatory cytokine response (6), to increase the risk of obesity, dementia and diabetes (7–10) and to compromise mood, performance and alertness (11, 12). Finally, both long (>9 h) and short (< 6 h) sleep durations are associated with increased risk of all-cause mortality (13, 14).

Especially the association between sleep and pain has been highlighted in the literature (15–20). Considering the biopsychosocial approach to pain (21–23), it seems logical that this relationship exists. However, the directionality of the sleep/pain association is not entirely clear and has been a progressively growing area of research: Early longitudinal studies were reviewed by Smith and Haythornthwaite in 2004, where 5 of 6 and 4 of 6 studies reported evidence for a pain sleep and a sleep pain directionality, respectively (20). Since then, more than twice as many prospective studies investigating the directionality of the sleep/pain association have emerged, and the collected evidence from more recent research utilizing longitudinal and micro-longitudinal research designs points toward a slightly greater effect of sleep on pain than pain on sleep (17). However, regardless of directionality, it is abundantly clear that sleep and pain strongly influence one another among the general population. What is less clear is the strength of this association within specific groups of the working population. Hospital workers constitute a population with a well-known high prevalence of musculoskeletal disorders (MSD). For instance, more than 36% of Danish nurses experience pain several times during a normal work week, and the annual prevalence of low back pain is 55% among this subgroup of the working population (24, 25). Given the stressful working environment and irregular working hours, it is not unlikely that sleep deprivation and increased sensitivity to pain will result in serious consequences when working with patients. Additionally, as is the case with sleep and pain, psychological stress often co-occurs with sleep deprivation (26). In fact, nurses have a high risk of developing emotional distress related to job stress; such as burnout, anxiety and depression, which will have detrimental effects on the physical and mental wellbeing (27, 28).

Therefore, this study aims to quantify the association between subjective measures of sleep and perceived pain intensity on a large population of hospital workers. Aside from being novel in investigating the sleep/pain relationship among this specific population of the workforce, we include correlations between sleep and stress as a secondary outcome. Although other potential risk factors—such as the musculoskeletal load during daily patient handlings (29)—still need to be investigated in more detail, the associations between vital lifestyle factors presented herein should be used as guidelines to direct future efforts with the goal of improving the overall wellbeing and working environment among hospital workers.

## Methods

### Study design and participants

This study is a cross-sectional questionnaire survey among 3,593 hospital workers from Danish hospitals, with 2,098 working as nurses. The survey seeks to elucidate the effect of work-environmental factors as well as different types of patient transfers and the use of assistive devices on the prevalence of back injury and low back pain, and includes 1-year follow-up, biweekly short questionnaires as well has technical measurements of muscle activity and body position during patient handlings (29).

The current study presents baseline analyses on the associations between stress/sleep and pain/sleep. Data was collected during the summer 2017 from hospitals in northern Denmark. As a way to achieve a generalized understanding of the health and working environment in Danish hospitals, the only inclusion criterion was that all participants were working at hospital. Following this, we did therefore not divide participants into subgroups based on occupation. Table 1 describes participant demographics and mean stress scores rated on Cohen's Perceived Stress Scale.

**Table 1:** Demographics, *N* = 3,593. CPS; Cohen's perceived stress.

	**Mean**	**(*SD*)**	**%**
Age (y)	45.8	(11.4)
BMI (kg/m^2^)	25.1	(4.7)
CPS (0–40)	12.5	(5.8)
**SEX**
Women			86
Men			14
**SMOKING**
Yes			9
No			91

### Outcome variable

#### Sleep

With the goal of achieving a broad indicator of overall sleep quality, 3 questions adapted from the Bergen Insomnia Scale regarding initiating & maintaining sleep, ease of awakening and overall tiredness during daytime, were asked:

How often during the previous 4 weeks have you…
a) …awoken during the night and having trouble going back to sleep?
b) …been feeling exhausted when waking up?
c) …been feeling tired during the day?

These questions have been used in the 2012, 2014, and 2016 rounds of the Danish Work Environment Cohort Study (DWECS), with questionnaires sent out to more than 50,000 people in the Danish workforce during each round. By using data previous published on this cohort (*n* = 7,883) we conducted a Pearson's correlation between the 3 questions used in this study and all 6 questions of the Bergen Insomnia Scale (PCC 0.94). Furthermore, we also performed an analysis on the correlation between the questions used herein and the remaining 3 questions of the scale (PCC 0.78) (30). Because of the high correlations presented we are confident in utilizing this simplified version as an indication of sleep quality.

The questions were rated on a 5-point Likert scale with items consisting of “never,” “rarely,” “sometimes,” “often,” and “always.” Based on the 3 questions the scores were then converted to a scale ranging from 0–100, with 0–50 and 50–100 indicative of poor and good sleep, respectively. This was done by allocating each item of the Likert scale a numeric score; ranging from 0 (“always”) to 100 (“never”), with 25-point intervals between scores.

Table 2 shows the distribution of poor and good sleep. Because not all participants replied to all questions, the exact number of replies varies.

**Table 2:** Distribution of sleep, pain and stress scores.

**Variable**		***N***	**%**
Poor sleep		1,919	57
Good sleep		1,432	43
No/low stress		1,212	37
Moderate stress		1,803	54
High stress		301	9
No/low pain		1,565	47
Moderate pain		1,568	48
High pain		175	5

### Predictor variables

#### Pain

As part of the questionnaire, the participants were asked to rate their average pain intensity within the previous 4 weeks for 9 body regions (neck, shoulder, upper back, lower back, elbows, hands/wrists, hips, knees and feet/ankles), on a visual numeric scale ranging from 0 to 10. The values for the 9 body parts were then averaged to represent a global pain score for the individual. Values 0 to 1 were characterized as “no/low pain”, whereas values 1.001 to 4 and 4.001 to 10 were characterized as “moderate pain” and “high pain,” respectively. Table 2 shows the distribution of no/low, moderate and high pain scores among the participants.

The numerical rating scale (NRS) is a simple technique for measuring a subjective experience. The NRS is valid, reliable and appropriate for use in clinical practices, and has been widely used to subjectively measure pain intensity. The NRS shows higher compliance rates, better responsiveness as well as higher sensitivity and applicability in comparison to the visual analog scale (VAS) (31, 32). Therefore, in this questionnaire study we used a modified NRS/VAS scale; consisting of a horizontally drawn line with numerical indications from 0 to 10. The participants answered by marking one of the numerical values on the line for each of the 9 body regions.

#### Cohen's perceived stress

The perceived stress scale by Cohen (referred to as Cohen's Perceived Stress Scale, CPSS) is a widely used measurement of subjective stress levels (36). Answers to questions on the scale are rated on a 5-point Likert scale, identical to the one mentioned previously to quantify sleep quality (ranging from “never” to “always”). Scores are then summed; with higher scores indicate a higher level of perceived stress. To ensure classification of the reference group, i.e., “no/low pain,” the following divisions were used: 0–10, 10.001–20, and 20.001–40 for low, moderate and high stress, respectively.

For the general population, scores of ~13 and above 20 indicate normative values and high stress, respectively. Table 2 shows the distribution of no/low, moderate and high stress scores among the participants.

### Statistics

SAS version 9.4 was used for all analyses. Associations between stress and pain (mutually adjusted predictors) and sleep (outcome) were modeled using binary logistic regression (Proc Genmod) controlling for age, sex (male/female), education, BMI (mass/h^2^) and smoking (yes/no).

Values are presented as Risk Ratios (RR) and 95% confidence intervals (CI).

## Results

A total of 3,593 hospital workers participated in this questionnaire survey. The number of missing answers on questions related to sleep, stress and pain was 249, 284, and 292, respectively. Table 2 shows the distribution of answers as absolute numbers (N) and percentages (%).

Among this population, the risk ratio of moderate stress (compared to no/low stress) on poor sleep was 1.27 (95% CI 1.26–1.29), whereas the risk ratio of high stress on poor sleep was 1.87 (95% CI 1.83–1.91). Similarly, for pain, the risk ratio of moderate pain (compared to no/low pain) on poor sleep was 1.18 (CI 1.16–1.19), whereas the risk ratio of a high pain score on poor sleep was 1.48 (CI 1.44–1.52) (Table 3).

**Table 3:** Risk ratio (RR) of stress and pain on risk of poor sleep.

**Variable**		**RR**	**CI**
Stress	Moderate vs. low	**1.27**	1.26	1.29
	High vs. low	**1.87**	1.83	1.91
Pain	Moderate vs. low	**1.18**	1.16	1.19
	High vs. low	**1.48**	1.44	1.52

*CI; 95% confidence interval. Adjusted for gender, age, education, BMI, and smoking; RR, Risk Ratio*.

## Discussion

This study shows that stress and pain are associated with increased risk of poor sleep in a population of 3,593 Danish hospital workers. Our results illustrate that both stress and pain influence the risk of poor sleep in a dose-response manner.

Interestingly, the association between stress and sleep was stronger than that between pain and sleep. High levels of perceived stress seem to be detrimental to the health of the individual not only as a consequence of the stress response itself, but also via an increased risk of poor sleep: Following only one night of sleep deprivation, participants from a recent well-controlled experimental study experienced increased cortisol levels as well as increased levels of subjective stress (26). Following this, prolonged high levels of stress may therefore negatively influence the workability of this population (33), as the effects of stress and poor sleep are likely to be additive over time.

Similar to the nature of pain and despite prevailing views in modern society, stress cannot be considered inherently bad. Acute episodes of stress, with the accompanying release of catecholamines and cortisol, serve evolutionary important functions in regards to the “fight or flight”-response. Cortisol, a potent and longer-lasting chemical, is produced in a daily pattern by the adrenal gland to deal with stressors (34). Under normal circumstances the body benefits from and is more than capable of handling acute increases in stress hormones. However, as with pain, when the stress-response becomes persistent without credible external cues, issues related to health and wellbeing are likely to develop as prolonged exposure to high cortisol levels leads to a number of unfortunate biological events such as low libido, temporary infertility, inflammation, weight gain, appetite changes and obesity (35, 34).

As a valid indicator of perceived stress, normative values [mean (SD)] for Cohen's Perceived Stress Scale are 12.1 (5.9) for men and 13.7 (6.6) for women (36, 37). Therefore, a score of approximately 13 should be considered average in the working population. With a perceived stress score of 12.5 (5.8) observed in the present study, this population of hospital workers does not constitute a unique situation but is likely to represent a trend in the general working population. Considering the strong dose-response relationship between stress and sleep, it seems vital to focus on one or both of these lifestyle factors as they are likely to influence each other bi-directionally. Following this, even slight improvements in stress and/or sleep parameters are likely to influence work ability and sickness absence positively among hospital workers (38).

Similar to the acute stress response, we require both sleep and pain for survival. However, persistent impairments to systems regulating sleep and pain will result in broad and pronounced negative impacts on health and well-being. Pain is a strong behavioral motivator that serves to protect the individual from harm. As a classic example, the danger signal that is sensed by the brain when accidentally touching a hot stove will cause a reflective withdrawal of the hand to avoid permanent tissue damage. This example serves as a reminder that pain itself is not to be considered a negative phenomenon, as it serves a vital evolutionary purpose. However, when the brain continues to produce pain even when the tissue damage has long since healed and the survival of the organism is no longer threatened, persistent pain—likely characterized and influenced by neuro inflammation and central sensitization—can develop (39). Within the population of Danish hospital workers—especially among nurses—it is likely that the high prevalence of musculoskeletal pain is an indicator of a system experiencing continuous threat due to various job-related stressors; e.g., mechanical overload, understaffed and odd working hours that might influence sleep negatively. This adds evidence of perceived threat to the individual and hence increase the likelihood of experiencing pain (40, 24).

In the current study, the participants were asked to rate their levels of perceived stress, pain and sleep quality during the previous 4 weeks. More than half of the participants rated their sleep as being poor, as well as their pain- and stress levels to be either moderate or high. Similar to the situation in other regions of the world regarding the working environment among hospital workers (27, 28), these numbers serve to highlight the notion that the wellbeing as well as the working environment of Danish hospital workers, especially among nurses, could be improved.

Whereas the stress response needs to be persistent over time in order to elicit detrimental effects on health and wellbeing, only minor disturbances in sleep are needed in order to experience negative consequences. Indeed, increased pain sensitivity after only 1 night of partial (4 h) sleep loss has been observed (3). Extending these findings, 1 night of total sleep deprivation has been shown to induce a state of generalized hyperalgesia and mood changes associated with increased anxiety levels (4). Luckily, these changes in pain sensitivity can readily be reversed with extended time in bed (41), which highlights one of the possible actions to easily counteract the consequences of sleep loss and poor sleep in general.

Another potent way to increase sleep quality and duration is to improve sleep hygiene; e.g., ensure a completely dark, cool and dimmed-light bedroom. Limiting the amount of blue light (i.e., light from electronic equipment) in the hours prior to bedtime has been shown to positively affect various sleep parameters including evening sleepiness, melatonin secretion, circadian rhythm as well as next-morning alertness (42). Furthermore, limiting light- and screen exposure prior to sleep is not only an easy and effective way to prevent sleep deprivation, it also has the potential to influence sleep related issues on a national scale: In a study involving 1,508 participants, 9 out of 10 reported using technological devices within 1 h before bedtime (43), indicating a great potential for improving sleep hygiene among the general population. In the context of hospital workers, especially during evening- and nightshifts, it is highly likely that the exposure to blue light will affect sleep and circadian rhythm negatively.

Taken together, it seems evident that stress, sleep and pain likely have an additive impact on the working environment of hospital workers. Furthermore, although not assessed in the current study, relatively small changes are likely to influence the health and well-being of this population by decreasing job-related stressors, and hence perceived threat.

## Strengths and limitations

The limitations of this study include the fact that we are unable to establish directionality between the associations between stress, pain and sleep. However, based on the cited literature and the clear dose-response relationships between these, they all very likely influence one another. Following this, because a broad range of factors are likely to influence overall sleep quality in an individual, the limitations of utilizing a simplified version of the Bergen Insomnia Scale should be noted and any practical implementation strategy should reflect this notion. Furthermore, the results cannot be generalized as they are specific to the working population of hospital workers; the majority being nurses. Clear strengths of the present study are the high number of participants and the establishment of a subjective global pain score based on 9 body regions. Therefore, rather than being specific to one type of pain or one body part, the values presented in this study represent an overall perceived pain score in a subpopulation of the workforce.

## Conclusion

Clear dose-response relationships exist between perceived stress and poor sleep as well as between pain intensity and poor sleep among hospital workers. The long-term consequences on health and workability of these associations need to be investigated in detail, but for now they should serve as indications as to where hospitals could intervene in order to improve the local working environment.

## Ethics statement

In accordance with Danish laws, ethical approval is not required when performing a questionnaire-study. The study is approved by the Danish Data Protection Agency (j. nr. 2015-41-4232).

## Author contributions

JV, MJ, JC, KJ, and LA contributed to the design of the study as well as analysis and interpretation of the results. JV drafted the manuscript while MJ, JC, KJ, and LA revised it critically before approving the content for publication.

### Conflict of interest statement

The authors declare that the research was conducted in the absence of any commercial or financial relationships that could be construed as a potential conflict of interest.
